# Oral health‐related quality of life and associated factors among older people in short‐term care

**DOI:** 10.1111/idh.12424

**Published:** 2019-12-18

**Authors:** Susanne Koistinen, Lena Olai, Katri Ståhlnacke, Anna Fält, Anna Ehrenberg

**Affiliations:** ^1^ School of Education, Health and Social Studies Dalarna University Falun Sweden; ^2^ Department of Public Health and Caring Sciences Family Medicine and Preventive Medicine Uppsala University Uppsala Sweden; ^3^ School of Medicine and Health School of Health Sciences Örebro University Örebro Sweden; ^4^ Dental Research Department Postgraduate Dental Education Center Örebro Sweden; ^5^ Clinical Epidemiology and Biostatistics School of Medical Sciences Örebro University Örebro Sweden

**Keywords:** older people, oral health, Oral health‐related quality of life, self‐perceived, short‐term care

## Abstract

**Objectives:**

It is well known that oral health status is associated with oral health‐related quality of life (OHRQoL) in the general population. The aim of this study was to describe and analyse OHRQoL among older people in short‐term care and its associated factors.

**Materials and Methods:**

This cross‐sectional study included 391 older people in 36 short‐term care units. Data were collected via clinical oral assessments, questions about self‐perceived oral and general health, Katz Index of Activities of Daily Living (Katz‐ADL) and the Revised Oral Assessment Guide (ROAG). OHRQoL was measured using the Oral Health Impact Profile (OHIP‐14). Multivariate logistic regression models were applied in the analysis.

**Results:**

Poor OHRQoL was reported by 34% of the older people. Associated factors were swallowing problems according to ROAG; quite poor/poor self‐perceived physical, psychological and oral health; and being a woman.

**Conclusions:**

There is an association between OHRQoL and older people's self‐perceived health according to the OHIP‐14. This indicates the importance of early detection of oral health problems in frail older people and to assess both oral health and swallowing problems among older people in short‐term care.

## 1 INTRODUCTION

Oral health is associated with people's well‐being and quality of life as it includes the ability to speak, smile, smell, taste, touch, chew, swallow and express emotions by facial expressions without pain and discomfort.^1^ Quality of life (QoL) has several definitions, and according to WHO, QoL is defined as a person's perceptions of their situation in life, the context of the culture in which they live and in relation to their goals, expectations, standards and concerns.^2^ In the context of health and disease, QoL is often referred to as health‐related quality of life (HRQoL),^3^ which is influenced by oral health. This is especially relevant for older people, because of the increase in general health problems and its association with oral health.^4^ Oral health‐related quality of life (OHRQoL)^5^ is a description of a person's perceived health, well‐being and quality of life related to oral conditions and function.^2^

One of the most widely used instruments to measure OHRQoL is the oral health impact profile (OHIP) and its shortened version (OHIP‐14).^6^ OHIP was developed to examine the impact of oral problems (ie problems with the teeth, mouth and dentures) on a person's daily life,^7^ and specifically among older people.^8^

Today, many older persons in Sweden have a high number of remaining natural teeth, and denture wearers are less common.^9^ The dominant oral diseases are dental caries and periodontal diseases. However, there are several factors that make older people particularly sensitive to these diseases, such as the influence of the ageing process on the immune system function, morbidities and medication which may reduce the salivary flow.^10^ Poor oral health status is prevalent among older people in special accommodations,^11^ and it is associated with malnutrition due to its effect on chewing and swallowing.^12^

Maintaining good oral hygiene becomes more challenging in old age,^10^ because of decline in abilities such as sight and mobility,^13^ cognition and functioning in activities of daily living (ADL).^10^ Thus, older people in need of care often have poor oral health and need help with their daily oral care.^14^

The description and measurement of oral health has been dominated by a biomedical approach. However, oral health assessment should be based on a holistic perspective, as a person may experience good oral health but still have clinical signs of caries or periodontal disease and vice versa.^15^ It is therefore necessary to consider both objective (clinical) and subjective (self‐rated experience) measures for assessment of oral health.^16^

Supporting a patient's well‐being goes beyond simply treating oral diseases, and therefore, patient‐oriented perspectives such as OHRQoL are important in order to improve the understanding of the relationship between oral health, general health and quality of life.^5^ To promote person‐centred care, a greater emphasis is needed on the assessment of OHRQoL.^17^

Many factors may affect older people's OHRQoL. Previous research has shown poorer OHRQoL among people with poor self‐rated health,^4^, ^18^ people dependent on support in their ADL,^19^ people with missing teeth,^20^ caries and periodontal disease,^21^ and poor self‐rated oral health.^22^ However, these relationships have not been established for older people in short‐term care.

In Sweden, the municipalities are responsible for home services, special accommodation and short‐term care for older people. Short‐term facilities provide nursing care for days to months for people that are recovering after a hospital stay, undergoing rehabilitation, respite care, palliative care or waiting for special accommodation placement.^23^ In short‐term care, oral care may be neglected due to the expected short care episodes. Because of limited research about oral care in this context and the effects on the older peoples QoL,^23^ this study can increase our knowledge about OHRQoL among older people in short‐term care.

To our knowledge, there have been no studies of OHRQoL among older people in short‐term care settings. More knowledge is needed in order to design effective oral health programmes to be able to improve OHRQoL. The aim of this study was to describe OHRQoL and identify its associated factors among older people in short‐term care.

## 2 MATERIAL AND METHODS

### 2.1 Design and setting

This descriptive cross‐sectional study was carried out within the framework of an ongoing Swedish research study in short‐term care: Swallowing Function, Oral Health, and Food Intake in Old Age (SOFIA).^24^ Short‐term care staff comprise licensed practical nurses, nurse aides, registered nurses and occupational therapists.^23^

A sample of 5 out of the total 21 Swedish regions was invited to participate, and all the heads of 19 social welfare services and unit managers provided informed consent. The study included 36 short‐term care units covering both rural and urban areas. All short‐term care units were selected by convenience, based on their geographical location, number of beds and estimated numbers of discharges per month.^24^

### 2.2 Participants

Older people admitted to the selected short‐term care units were eligible for the study. Inclusion criteria were being ≥65 years, having stayed at the short‐term care unit for at least 3 days, being able to understand Swedish and having sufficient cognitive ability (based on patient records and judged by registered nurses) to give informed consent and to participate in data collection. Persons receiving end‐of‐life care were excluded.^24^ Of the 931 people assessed for eligibility, 477 (51%) did not meet the inclusion criteria. The reasons for exclusion were palliative care (n = 61), insufficient cognitive capacity (n = 309) and being younger than 65 years, admitted for less than 3 days or being unable to communicate in Swedish (n = 107). The remaining 454 were all invited to participate, and 63 (14%) declined, resulting in a total sample of 391 older people.

### 2.3 Instruments

Data were collected via clinical oral assessment including questions about self‐perceived oral and general health, the Katz Index of Activities of Daily Living (Katz‐ADL), the Revised oral assessment guide (ROAG) and the OHIP‐14.^24^

#### 2.3.1 Clinical oral assessment

The oral assessment included number of natural teeth, presence of partial or full dentures, presence of bridges and implants, and need for dental care. The registered dental hygienists (RDHs) assessed need for dental care based on both the clinical assessment and ROAG. One question was asked about the person's ability to brush their teeth, with response options 1=“yes, completely,” 2=“receive some help” and 3=“no, receive help entirely”.^24^

#### 2.3.2 Self‐rated oral health and general health

Self‐ratings of oral, physical and psychological health were assessed on a 5‐point scale (1= “poor,” 2= “quite poor,” 3=“neither good nor poor,” 4= “quite good” and 5=“very good”).^25^

#### 2.3.3 Assessment of functional status

Self‐care ability was assessed using the Katz‐ADL,^26^, ^27^ which summarizes a person's overall performance concerning six functions: bathing, dressing and undressing, going to the toilet, mobility, controlling bowel and bladder, and food intake. Performance in each activity is graded from A to G: A = independent in all functions, B = dependent on help in one activity, C = dependent in two activities, D = dependent in three activities, E = dependent in four activities, F = dependent in five activities and G = dependent in all six activities.^26^

#### 2.3.4 Revised oral assessment guide

Oral health status was assessed using the revised oral assessment guide‐Jönköping (ROAG‐J).^28^ This is an adapted version of the ROAG, designed for use by nursing staff to detect problems related to mouth, teeth and dentures in older people.^11^, ^29^ Nine items are included as follows: voice, lips, mucous membranes, tongue, gums, teeth, dentures, saliva and swallowing (eg pain or dryness when swallowing saliva).^28^, ^29^ Voice and lips are graded by conversing with the person and observing; mucous membranes, tongue, gums, teeth and dentures are graded via observation using a mouth mirror and flashlight; and swallowing sensation is graded by observation when the person is asked to swallow.^11^ Each category is graded on a three‐point scale (1=“healthy,” 2=“moderate oral health problem” and 3=“severe oral health problem”).^11^, ^29^ The total score ranged from 8 (healthy/without oral problems) to 24 (severe oral health problems/with oral problems).

#### 2.3.5 Oral health impact profile (OHIP‐14)

Oral health‐related quality of life (OHRQoL) was measured using the OHIP‐14, which includes 14 items to capture seven dimensions: functional limitation (trouble pronouncing words and altered sense of taste), physical pain (painful aching in mouth and discomfort when eating), psychological discomfort (self‐consciousness and being tense), physical disability (unsatisfactory diet and interrupted meals), psychological disability (difficulty relaxing and feeling embarrassed), social disability (irritability and difficulty performing daily tasks) and handicap (finding life less satisfying and being unable to function).^8^ Each item is assessed using the same question—“How often during the last month have you experienced the following situation because of problem with your teeth, mouth, dentures or jaw?”—answered on an ordinal scale from 0 to 4:0=“not applicable” or “never,” 1=“hardly ever,” 2=“occasionally,” 3=“often” and 4=“very often.” A total score (range: 0‐56) is obtained by adding up the points for the individual questions,^30^ with higher scores indicating poorer OHRQoL.^31^

### 2.4 Procedure

The heads of social welfare of elderly care and short‐term care units in each municipality were contacted to provide information and request approval of the study. The registered nurse in charge at each unit made an initial assessment of the older persons to identify those who fulfilled the inclusion criteria and could be approached for providing informed consent to participate in the study. Both oral information and written information about the study were provided. Data regarding participants’ age, gender, educational level and medical diagnoses were collected from patient records and self‐reports. A licensed practical nurse or the registered nurse answered questions concerning the older person's self‐care ability. The research assistants (eight RDHs and one speech language pathologist) collected self‐reported questionnaire data by interviewing the participants, and the RDHs carried out a clinical assessment and ROAG assessment using a mouth mirror and a flashlight.^24^

Data were collected from October 2013 to January 2016. In order to achieve sufficient power for the intervention of the larger SOFIA study,^24^ one more region was included resulting in a prolonged data collection. Ethical principles were followed, including informed consent, confidentiality and the right to withdraw from participation at any time without giving a reason. The RDHs informed the participants and the responsible nurse about the need to contact dental care for treatment if any severe oral health problem was detected. The study was approved by the Regional Ethical Review Board in Uppsala, Sweden (ref: 2013/100, 2013‐04‐03), and was carried out according to the ethical principles of the Helsinki Declaration.^32^

### 2.5 Statistical analyses

Descriptive results are presented with frequencies and percentages. Thirteen respondents answered all the questions in the OHIP‐14 as “not applicable” or left the questions unanswered, and twelve had left four or more questions unanswered, which was regarded as an internal non‐response. The number of respondents to OHIP‐14 was therefore 366. For the descriptive results, the answers in OHIP were divided into three categories (0=“never,” 1=“hardly ever” and “occasionally,” and 2=“often” and “very often”). The cut‐off value was chosen to be 7 and 8 since 8 is equating to two items at the “very often” level. For the analyses using chi‐square test and regression analyses, the OHIP score was dichotomized as 0 = OHIP score ≤ 7 and 1 = OHIP score ≥ 8 (equating to two items at the “very often” level).^4^ The question about performing oral self‐care was categorized as 1=“yes, completely” and 2 = either “receive some help” or “no, receive help entirely.” The self‐perceived health questions were divided into three categories: 1= “quite good” or “very good,” 2= “neither good nor poor,” and 3=“quite poor” or “poor.” The Katz‐ADL index was divided into three categories: A = independent, B–D = partly dependent and E–G = completely dependent.^27^, ^33^

Multivariate logistic regression analyses were used to calculate odds ratios (ORs) and 95% confidence intervals (CIs) with OHIP score (0 = ≤7, 1 = ≥8) as dependent variable. The first regression model included the following items from ROAG as independent variables: voice, lips, mucous membranes, tongue, gums, teeth and dentures (merged into one), saliva and swallowing. The merging of teeth and dentures resulted in eight items with a total score ranging from 8 (healthy) to 24 (severe oral health problems). The second model included the total ROAG score (0=“without oral problems” [score = 8], 1=“with oral problems” [score = 9‐24]) as independent variable.^14^ A third model was fitted with various demographic and clinical characteristics as covariates. All models were adjusted for sex, age and education. Data were checked for multicollinearity. The significance level was set at *P* ≤ .05.

## 3 RESULTS

The results are based on 391 older people from 36 short‐term care units in five Swedish regions: 209 (53%) women and 182 (47%) men. Of these, 214 (55%) were 65‐84 years old and 177 (45%) were 85‐100 years old (mean age = 82.9). The main medical diagnoses were stroke 22%, musculoskeletal disease/locomotor disorder 22% and mild cognitive impairment 12%; and 53% had three or more medical diagnoses.

### 3.1 Clinical oral assessment

Table 1 shows that 43% had 20‐32 remaining teeth and 35% had removable dentures, and 41% were assessed as having a need for dental care. Nineteen per cent received some or total help with oral self‐care, and 81% performed oral self‐care themselves.

**Table 1:** Characteristics, oral status, self‐rated oral and general health and functional status of older people in short‐term care (n = 391)

Characteristics of the older people in short‐term care (n = 391)			
	Men n = 182 (%)	Women n = 209 (%)	Total n = 391 (%) *
Age, years			
65‐84	113 (62)	101 (48)	214 (55)
85‐100	69 (38)	108 (52)	177 (45)
Education			
Compulsory school	104 (58)	147 (71)	251 (65)
Upper secondary school	56 (31)	43 (21)	99 (26)
University	20 (11)	16 (8)	36 (9)
Oral status of the older people			
Number of teeth			
0	32 (18)	42 (20)	74 (19)
1‐19	69 (38)	79 (38)	148 (38)
20‐32	80 (44)	87 (42)	167 (43)
Removable dentures (full or partly)			
Yes	63 (35)	72 (35)	135 (35)
Bridges			
Yes	55 (30)	78 (38)	133 (34)
Implants			
Yes	16 (9)	17 (8)	33 (9)
Need for dental care			
Yes	68 (40)	80 (41)	148 (41)
Perform oral self‐care			
Yes, completely	134 (75)	176 (86)	310 (81)
Receive some help/receive help entirely	45 (25)	29 (14)	74 (19)
Self‐rated oral health and general health			
Self‐perceived oral health			
Very good/quite good	123 (69)	141 (68)	264 (69)
Neither good nor poor	28 (16)	30 (15)	58 (15)
Quite poor/poor	27 (15)	35 (17)	62 (16)
Self‐perceived physical health			
Very good/quite good	103 (58)	96 (47)	199 (52)
Neither good nor poor	26 (14)	34 (16)	60 (15)
Quite poor/poor	50 (28)	76 (37)	126 (33)
Self‐perceived psychological health			
Very good/quite good	115 (65)	118 (58)	233 (61)
Neither good nor poor	31 (17)	52 (25)	83 (22)
Quite poor/poor	32 (18)	35 (17)	67 (17)
Functional status			
Katz‐ADL			
A	14 (8)	14 (7)	28 (7)
B‐D	61 (34)	104 (50)	165 (43)
E‐G	104 (58)	88 (43)	192 (50)

* Numbers do not add to total due to missing data, with a range of 363‐391 respondents.

### 3.2 Self‐rated oral health and general health

Concerning self‐perceived health, 69%, 52% and 61%, respectively, perceived their oral, physical and psychological health as quite good/very good.

### 3.3 Functional status (Katz‐ADL)

A total of 50% were dependent on help with at least four ADL activities (E‐G). There were some differences between men and women. More men than women received some or entire help in performing daily oral care (*P* = .006), and dependence on help with 1‐3 ADL activities was more common among women (*P* = .005).

### 3.4 Oral health based on ROAG

The oral health in terms of the ROAG is shown in Table 2. The most frequent oral health problems were related to teeth and dentures. Visible coating or food debris locally/coating, food debris generally or broken teeth was found among 57%, and coating or food debris was present in 57% of the denture wearers. Twenty‐four per cent had insignificant or pronounced swallowing problems. Overall, oral problems [score = 9‐24] were identified in 297 (77%) older people.

**Table 2:** Oral health based on the Revised Oral Assessment Guide—ROAG (n = 390)

Oral health based on clinical assessment using the Revised Oral Assessment Guide—ROAG (n = 390)			
Item Category	Grade 1 Findings n (%)	Grade 2 n (%)	Grade 3 n (%)
Voice*	Normal *252 (65.1)*	Dry, hoarse, smacking *112 (28.9)*	Difficult to speak *23 (6.0)*
Lips*	Smooth; bright red; moist *322 (83.4)*	Dry, cracked, sore corners of the mouth *62 (16.1)*	Ulcerated, bleeding *2 (0.5)*
Mucous membranes*	Bright red; moist *325 (85.8)*	Red; dry or areas of discoloration, coating *52 (13.7)*	Wounds, with or without bleeding, blisters *2 (0.5)*
Tongue*	Pink, moist with papillae *303 (78.7)*	No papillae, red, dry coating *79 (20.5)*	Ulcers with or without bleeding, blistering *3 (0.8)*
Gums*	Light red and solid *243 (71.7)*	Swollen, reddened *93 (27.2)*	Spontaneous bleeding *6 (1.7)*
Teeth*	Clean; no visible coating, food debris *137 (42.8)*	Coating or food debris locally *146 (45.6)*	Coating, food debris generally or broken teeth *37 (11.6)*
Dentures*	Clean; works *53 (39.0)*	Coating or food debris *77 (56.6)*	Not used or malfunctioning *6 (4.4)*
Saliva*	Glides easily *304 (78.4)*	Glides sluggishly *78 (20.1)*	Does not glide at all *6 (1.5)*
Swallowing*	Unimpeded swallowing *287 (76.3)*	Insignificant swallowing problems *66 (17.6)*	Pronounced swallowing problems *23 (6.1)*

* Numbers do not add to total due to missing data, with a range of 3‐28 respondents.

The number and percentages of older people who answered the questions in OHIP‐14 are presented in Table 3. A majority reported that they had experienced no functional limitation due to problems related to teeth, mouth, dentures or jaw during the past month. Twenty‐seven per cent experienced functional limitations such as altered perception of taste hardly ever or only occasionally, 22% reported physical disability in the form of an unsatisfactory diet, 14% often/very often experienced difficulty performing daily tasks and 12% experienced that life in general was less satisfying due to oral health problems. There were no significant differences between men and women, except for psychological disability; women had more often or very often (*P* = .006) been embarrassed due to problems related to teeth, mouth, dentures or jaw.

**Table 3:** Numbers (n) and percentages of older people (n = 366) in short‐term care with and without oral health problems

OHIP‐14 Dimensions and items	Never n (%)	Hardly ever/occasionally n (%)	Often/very often n (%)
Functional limitations			
Trouble pronouncing words	259 (71)	69 (19)	36 (10)
Altered sense of taste	225 (62)	96 (27)	40 (11)
Physical pain			
Painful aching in mouth	250 (68)	100 (27)	17 (5)
Discomfort when eating	247 (67)	98 (27)	21 (6)
Psychological discomfort			
Self‐consciousness	301 (82)	52 (14)	16 (4)
Feeling tense	300 (81)	53 (14)	18 (5)
Physical disability			
Unsatisfactory diet	280 (75)	83 (22)	9 (3)
Interrupted meals	306 (83)	54 (14)	10 (3)
Psychological disability			
Difficulty relaxing	306 (82)	52 (14)	16 (4)
Feeling embarrassed	326 (88)	31 (8)	14 (4)
Social disability			
Irritability	299 (81)	60 (16)	9 (3)
Difficulty performing daily tasks	291 (79)	26 (7)	52 (14)
Handicap			
Finding life less satisfying	270 (73)	56 (15)	45 (12)
Being unable to function	304 (82)	42 (11)	25 (7)

### 3.5 Factors associated with oral health‐related quality of life

The median OHIP score was 4 (interquartile range: 0‐10). A total of 241 (66%) reported lower OHIP scores (≤7), and 125 (34%) reported higher scores (≥8). The univariate analysis showed that 100 (39%) of older people with oral problems according to ROAG had poor OHRQoL (OHIP scores ≥ 8) and 19 (25%) of those without oral problems had poor OHRQoL (*P* = .026). Among those who perceived their oral health as quite poor/poor oral health, 30 (50%) had poor OHRQoL and 69 (28%) of those with very good/quite good oral health had poor OHRQoL (*P* < .001). Of those who perceived their physical health as quite poor/poor, 57 (48%) had poor OHRQoL and the corresponding number for those with very good/quite good physical health was 49 (26%) (*P* < .001). Twenty‐seven (44%) of older people who perceived their psychological health as quite poor/poor had poor OHRQoL compared with 64 (29%) for those with very good/quite good psychological health (*P* = .015). Eighty‐one (41%) of all women had poor OHRQoL, and the corresponding number for men was 44 (26%) (*P* = .002). There were no significant relationships between OHIP score and age, education, performing oral self‐care, number of teeth, removable dentures, implants, bridges and need for dental care.

The regression model, including all nine ROAG items as independent variables, revealed that older people with swallowing problems were 5.4 times more likely to have poor OHRQoL compared to those with no swallowing problems (OR: 5.43; 95% CI: 2.80‐10.55). Other oral problems were not significantly related to OHRQoL.

Table 4 presents the adjusted ORs for having poor OHRQoL (OHIP score ≥ 8). Men were 60% less likely than women to report poor OHRQoL (OR: 0.41; 95% CI: 0.25‐0.70). The more unsatisfied older people were with their oral health, the higher was the likelihood of having poor OHRQoL. The ORs for “neither good nor poor” oral health and “very/quite poor” oral health compared with “very/quite good oral health” were 1.95 (95% CI: 1.01‐3.78) and 2.95 (95% CI: 1.50‐5.78), respectively.

**Table 4:** Multivariate logistic regression with OHIP as dependent variable (0 = OHIP score ≤ 7, 1 = OHIP score ≥ 8), divided by various demographic and clinical characteristics of older people (n = 366) in short‐term care

	Adjusted model OR (95% CI)	p
Gender		
Female	1.00 (ref)	
Male	0.41 (0.25‐0.70)	**<.001**
Age (continuous)	0.84 (0.50‐1.41)	.514
Education		
Compulsory school	1.00 (ref)	
Upper secondary school	1.64 (0.92‐2.92)	.096
University	0.63 (0.23‐1.74)	.375
Number of teeth		
0	1.00 (ref)	
1‐19	0.70 (0.35‐1.38)	.291
20‐32	0.92 (0.47‐1.83)	.818
Clinical assessment (ROAG)		
Without oral problems	1.00 (ref)	
With oral problems	1.87 (0.99‐3.54)	.055
Perform oral self‐care		
Yes, completely	1.00 (ref)	
Receive some help/entirely	1.84 (0.96‐3.54)	.068
Oral health		
Very good/quite good	1.00 (ref)	
Neither good nor poor	1.95 (1.01‐3.78)	**.048**
Quite poor/poor	2.95 (1.50‐5.78)	**.002**
Katz‐ADL		
A	1.00 (ref)	
B–D	1.40 (0.43‐4.28)	.607
E–G	0.97 (0.30‐3.11)	.955
Nagelkerke's Pseudo *R* ^2^	.151	

Note: Values in bold indicates statistical significant

Older people who have oral problems based on ROAG tend to more often report poor OHRQoL compared to those with no oral problems, although not significant based on a ≤0.05 level (*P* = .055). This also applies to those who received some or complete help with tooth brushing compared to those without help (*P* = .068).

## 4 DISCUSSION

In this study, we described OHRQoL and identified factors associated with OHRQoL among older people in short‐term care. The results show that OHRQoL is associated with older people's self‐perceived health, which also been shown in previous studies.^4^, ^18^, ^22^ Most participants reported good OHRQoL, but one third did not. More than half reported they had experienced physical pain (painful aching in the mouth and discomfort when eating), and about half reported functional limitations (trouble pronouncing words and an altered sense of taste). Factors associated with poor OHRQoL were being a woman, having oral problems according to ROAG and perceiving quite poor/poor physical, psychological and oral health. Those with swallowing problems (measured by ROAG) were five times more likely to have poor OHRQoL, which has never been studied before in this care context. It is important to have in mind that the ROAG assessment of swallowing sensation is graded via observing when the person is asked to swallow,^11^ and not via swallowing capacity test.^24^ Women were more likely than men to report poor OHRQoL, which is consistent with another study of OHRQoL among older people.^34^

The fact that many people in this study reported good OHRQoL may be because older people are more likely to consider minor or even severe oral health problems as less disturbing at this point in their lives because of other general health problems, and therefore express greater satisfaction with their oral health.^35^ Another explanation might be that 43% of the older people had as many as 20‐32 remaining teeth, a recent study found association between few remaining teeth and poor OHRQoL.^20^ The most frequently experienced oral problems associated with OHRQoL were painful aching in the mouth and discomfort when eating; this result is consistent with previous findings in a Norwegian study of people aged 68‐77 years.^36^

Although oral problems according to ROAG were identified in about three quarters of the participants, this was not significantly related to their OHRQoL. One explanation for no relation seen between oral health problems and OHRQoL may depend on where the cut‐off was drawn between those with and without oral health problems. Perhaps the result would have been different if the cut‐off had been done at those with most pronounced oral problems. That dichotomization was not done because there were too few individuals in the group with most pronounced oral problems. Another explanation may be that there are discrepancies between professionally measured oral health and self‐reported OHRQoL, especially among older people.^37^ This also indicates that older people may not perceive their oral health problems to have a significant impact on their OHRQoL due to other general health problems.^35^ It is important to include both a holistic perspective and a biomedical perspective,^15^ and to consider both objective (clinical) and subjective (self‐rated experience) measures for assessment of oral health.^16^ Therefore, a person‐centred care where the persons’ whole situation is taken into account in the assessment of oral health and early detection of oral health problems in frail patients or patients who are at risk of rapid deterioration is important.

We also found that the less satisfied the older persons were with their oral health, the higher their likelihood of having poor OHRQoL, which again has been reported in previous studies among older people.^18^, ^36^ Those who perceived their physical health as quite poor/poor in this study also had higher OHIP scores, which indicate associations between general health and oral health.

Participants with swallowing problems were five times more likely to have poor OHRQoL compared with those without such problems. The method of grading swallowing problems in ROAG does not reveal whether the swallowing problem is related to lack of saliva, dysphagia or other causes as chewing functions and neurological issues. Older peoples swallowing difficulties could also be related to having few teeth and occluding pair of teeth, which in turn affects chewing ability.^38^ Another important aspect is that one of the main diagnoses was stroke, and it is commonly known that stroke is associated with dysphagia.^39^ This indicates the need of good collaboration between healthcare staff and speech language pathologist in this care setting.

For many, tooth cleaning is a part of daily hygiene, and those who find it difficult to perform tooth brushing may feel that their mouth is unclean, which can cause distress or discomfort.^40^ A study of older people receiving home‐care nursing in Norway found poorer OHRQoL among those who had problems with tooth brushing, indicating the importance of adequate support. Problems with tooth brushing would decrease if assessment of older people's ability to perform oral hygiene and the need for assistance with oral care formed part of the regular assessments.^41^

Only about one fifth of the older people reported receiving some or complete help with oral self‐care. However, the clinical assessment showed that the most frequent oral health problems were coating, food debris and broken teeth, indicating that many of the older people needed assistance with oral self‐care. Since there were no observations about what kind of dental hygiene equipment the older people had available, we do not know whether they had access to their regular equipment during their stay in short‐term care. About half of the participants were dependent on help with at least four ADL activities, which could indicate a need for help in performing oral care. Dependency in ADL activities was not significantly related to self‐rated OHRQoL. The finding of no correlation between poor OHRQoL and ADL dependency could be explained by the impact of other disabilities and health problems, or it could be because oral self‐care is not included in ADL assessment.^18^ Conversely, a recent study found that dependence in ADL was associated with poor OHRQoL, which indicates that older people dependent in ADL also have difficulty in performing oral self‐care.^19^

Among those who perceived poor OHRQoL, more than a third had clinically assessed oral problems according to ROAG, and the most common problem was visible coating or food debris, and broken teeth. This indicates that if daily oral care was improved among older people, their OHRQoL could increase. In order to make oral care a part of the daily hygiene routine and meet each patient's needs, person‐centred care is of importance.^42^ A recent study with person‐centred, evidence‐based oral care programme in nursing homes showed significant improvement in oral hygiene outcomes.^43^

Since this study shows associations between OHRQoL and older people's self‐perceived health, there is a need to develop an interdisciplinary teamwork within elderly care. This requires teamwork among various professionals, including dental professionals, since good oral health is essential for healthy ageing.^44^ Different types of staff, including dental staff, should work together to share expertise, knowledge and skills in patient care.^45^ Systematic reviews show that inter‐professional teamwork has positive effects on older patients’ outcomes in hospital care.^46^ More knowledge is needed about older people's experience of their oral health and how this is related to their perception of overall health.

### 4.1 Methodological considerations

Methodological and ethical problems can be challenging in studies on older people in short‐term care.^47^ The fact that 477 of the eligible persons were excluded from the present study shows that many older people were too frail to participate. Since more than 50% of the eligible persons did not meet the inclusion criteria, the results of this study are not fully representative of the population of older people in short‐term care, for example, were persons in end‐of‐life care excluded, since they were judged as being too frail. Oral health and oral care are important for persons in end‐of‐life care but that was not within the scope of this study. The generalizability of the study may be hampered by the convenience selection of short‐term care units. However, the selection provided a good variation both geographically and in size of the settings. Because it was considered that answering questionnaires might be exhausting, the research assistants read the questions aloud in order to facilitate answering for the respondents and avoid misunderstandings. On one hand, this might have led to more accurate responses and fewer drop‐outs; but on the other hand, the research assistants’ support in reading and interpreting the questions might have introduced a risk of bias. The research assistants who performed the clinical oral assessments were registered dental hygienists with relevant clinical experience in communicating with older people and assessing oral health, which is a strength. All research assistants and the research group met regularly to ensure agreement and consistency in assessments. The ROAG is a screening tool developed for nursing staff in order to detect oral health problems among older people. ROAG does not include all oral health factors which are a limitation since it has been used in this study as an outcome measure. The use of ROAG was supplemented by obtaining other clinical data such as number of teeth, chewing surfaces, dental replacements and measurements of swallowing capacity. Generalizability was strengthened in that older people were included from 36 different short‐term care units and the units were located in five different regions, representing both rural and urban areas of Sweden.

## 5 CONCLUSIONS

Many old people in short‐term care are frail with multiple disorders and diseases involving extensive care needs, including need for support with oral care. As there is an association between OHRQoL and older people's self‐perceived health, early detection of oral health problems in frail old people is important. To improve oral care, it is necessary to develop teamwork among various professionals, including dental professionals, since good oral health is essential for healthy ageing.

## 6 CLINICAL RELEVANCE

### 6.1 Scientific rationale for study

Previous research has revealed many factors associated with older people's OHRQoL. This study contributes with new knowledge regarding oral health, OHRQoL and related factors among older people in short‐term care.

### 6.2 Principal findings

Our results confirmed association between OHRQoL and swallowing problems, poor self‐perceived physical, psychological and oral health.

### 6.3 Practical implications

To improve older people's general quality of life, well‐being and OHRQoL, ROAG should be complemented with a method for detecting the cause of swallowing problems. Interdisciplinary teamwork between health and dental professionals and more person‐centred approaches need to be developed to improve oral care and promote healthy ageing.

## CONFLICT OF INTEREST

The authors declare no conflict of interests.
